# Increased heart rate functions as a signal of acute distress in non-communicating persons with intellectual disability

**DOI:** 10.1038/s41598-021-86023-6

**Published:** 2021-03-19

**Authors:** Emilie Kildal, Kristine Stadskleiv, Elin S. Boysen, Tone Øderud, Inger-Lise Dahl, Trine M. Seeberg, Svein Guldal, Frode Strisland, Cecilie Morland, Bjørnar Hassel

**Affiliations:** 1 Department of Neurohabilitation, Oslo University Hospital, Oslo, Norway; 2 Department of Clinical Neurosciences for Children, Oslo University Hospital, Oslo, Norway; 3 Department of Special Needs Education, University of Oslo, Oslo, Norway; 4 SINTEF Digital, Oslo, Norway; 5 Oslo Municipality, Nordstrand, Oslo Norway; 6 Norwegian Farmers’ Association, Oslo, Norway; 7 Department of Behavioral Science, Oslo Metropolitan University, Oslo, Norway; 8 Department of Pharmacy, University of Oslo, Oslo, Norway; 9 Institute of Clinical Medicine, University of Oslo, Oslo, Norway

**Keywords:** Neurological disorders, Neurology, Risk factors, Signs and symptoms

## Abstract

Intellectual disability (ID) affects approximately 1% of the population. Some patients with severe or profound ID are essentially non-communicating and therefore risk experiencing pain and distress without being able to notify their caregivers, which is a major health issue. This real-world proof of concept study aimed to see if heart rate (HR) monitoring could reveal whether non-communicating persons with ID experience acute pain or distress in their daily lives. We monitored HR in 14 non-communicating participants with ID in their daily environment to see if specific situations were associated with increased HR. We defined increased HR as being > 1 standard deviation above the daily mean and lasting > 5 s. In 11 out of 14 participants, increased HR indicated pain or distress in situations that were not previously suspected to be stressful, e.g. passive stretching of spastic limbs or being transported in patient lifts. Increased HR suggesting joy was detected in three participants (during car rides, movies). In some situations that were previously suspected to be stressful, absence of HR increase suggested absence of pain or distress. We conclude that HR monitoring may identify acute pain and distress in non-communicating persons with ID, allowing for improved health care for this patient group.

## Introduction

Intellectual disability (ID) is a neurological condition that affects approximately 1% of the population^1, 2^. ID is highly heterogeneous both phenotypically and in terms of causality. It may be an acquired condition, e.g. through cerebral hypoxia, ischemia, or infection early in life^3–6^, or it may have a genetic cause, most often a de novo mutation^7^. The diagnosis implies IQ below 70, reduced adaptive skills (manifesting as difficulties in managing everyday life, school, work, interpersonal relations, etc.), and occurrence of the condition before the age of 18^8–10^. In the majority of cases, the condition is evident early in life. Approximately 5% of persons with ID have severe ID (IQ: 20–34) or profound ID (IQ < 20)^11, 12^. These patients are entirely dependent on their caregivers, but intellectual shortcomings limit their ability to communicate with them. Communication may be further limited if the ID is compounded by autism or by cerebral palsy that affects control of muscles involved in speech, gesticulation, and grimacing. Some of these individuals may be essentially non-communicating.

Persons with ID are prone to painful conditions, e.g. low impact fractures^13^ or various abdominal conditions^14, 15^. A series of studies has made it clear that persons with ID are at least as sensitive to pain as the general population^16–20^ but signs of pain may be lacking or overlooked by caregivers^21–23^. Structured interviews, questionnaires, and checklists aimed at caregivers have been developed to identify behavioral signs of pain in persons with ID^24–26^. However, even caregivers who are experienced observers may fail to identify painful conditions in non-communicating persons with ID^17, 27, 28^. Thus, there is a need to explore additional measures of pain detection that are applicable to a real-world situation.

Increased heart rate (HR) is an autonomic response to acute pain and distress^29–31^ and to happiness and excitement^32, 33^, as well as a response to postural changes and muscle work. Therefore, HR monitoring—combined with observations of situational context and behavior—could be of use for non-communicating persons with ID as a means of conveying that they experience acute pain or distress.

This proof of concept study aimed to assess the possibility of using HR monitoring to identify acute pain or distress in non-communicating persons with ID in everyday situations. To this end, we monitored HR to see if increases in HR could identify potentially painful or distressing situations. Importantly, we used HR to detect participants’ perceived pain, not to identify pain as a sign of impending tissue damage, which would have to be detected by other means.

## Results

### Participant characteristics

The group of participants was heterogeneous with respect to the cause of their ID, but they all had communication difficulties that rendered them essentially non-communicating; some also had severe cerebral palsy or autism (Table 1). Some participants used a few words or phrases of uncertain meaning; others had no spoken language at all. Most participants had some form of behavior that was interpreted by caregivers as conveying information about preferences and dislikes, pain and pleasure. This behavior ranged from smiles and grimacing with an emotional value that seemed intelligible to caregivers, to more personal or idiosyncratic signs or movements whose interpretation was less obvious. In their response to the Inventory of Potential Communicative Acts^24^, caregivers mostly agreed among themselves on how to interpret the various behaviors of the participants (data not shown).

**Table 1:** Clinical characteristics of patients.

Patient	Communication	Self injury/aggression	Comorbidity	Medication	ID grade	Mode of transportation
F59	Laughs, screams for hours, bites hand	Self injury	CP, epilepsy Diabetes type 1 Scoliosis	Insulin Carbamazepine Ramipril	S	Wheelchair
F31	Smiles, facial expression of fear, reaches for objects	None	Rett syndrome Epilepsy Osteoporosis Scoliosis	Carbamazepine Levetiracetam Valproate	S	Wheelchair
F18	Screams, bites	Both	Autism Epilepsy Scoliosis	Carbamazepine Clonazepam Aripiprazole Omeprazole	S	Fully ambulant
M54	Speaks a few short phrases	Aggressive behavior	Diabetes type 1 Polyneuropathy	Insulin Gabapentin	S	Fully ambulant
F58	Some signs with her hands. Murmuring	Self injury	Epilepsy Hip dysplasia	Phenobarbital	S	Wheelchair
M62	Facial expression, sounds	None	Scoliosis Hip fracture	–	S	Ambulant + wheelchair
F 36	Smiles, moaning, screams, grimacing	None	CP/quadriplegia Tuberous sclerosis Hiatal hernia	Valproate Acetylcysteine Methenamine	S	Wheelchair
M48	Hand or head movements	Self injury	CP Spasticity Epilepsy	Lacosamide Levetiracetam Topiramate Phenobarbital	P	Wheelchair
M 37	A few words, body language	Self injury	Epilepsy Hypertension Spina bifida	Candesartan Amlodipine Lamotrigine Levetiracetam	S	Wheelchair
F56	Facial expression, laughter, shouting	None	CP Scoliosis	Baclofen Pantoprazole	Mo	Wheelchair
M27	Smiles, body movements Some signs	Both	Epilepsy Autism	Valproate Levetiracetam Clobazam Rufinamide	P	Fully ambulant
F22	Eye movement, vague facial expressions, howling	None	Rett syndrome Epilepsy Scoliosis	Lamotrigin Valproate	S	Wheelchair
F14	Facial expressions, some body movements	Both	CP, Epilepsy Impaired vision Insomnia	Zonizamid Clobazam Lamotrigin Valproate	S	Wheelchair
M18	Some facial expression, repetitive sounds, movement of head and arms	None	Hurler syndrome Epilepsy Paraplegia	Valproate Melatonin	S	Wheelchair

Sex and age (first column), modes of expession, presence of aggressive or self-injurious behavior, comorbidities, medication, degree of intellectual disability, and mode of transportation in 14 non-communicating persons with ID. Abbreviations: CP: cerebral palsy, F: female, ID: intellectual disability, M: male, Mo: moderate ID (IQ 35–49), S: severe ID (IQ 20–34). P: profound ID (IQ < 20).

Cognitive assessment confirmed that the participants had severe or profound ID, except one woman with severe cerebral palsy whose level of comprehension was equivalent to moderate ID (IQ 35–40). Several participants had restricted mobility, used a wheelchair, and were dependent on being lifted from bed to chair or from one chair to another, often with the use of an electric patient lift (Table 1). Three participants were fully ambulant. Most participants had comorbidities such as cerebral palsy, autism, epilepsy, diabetes, osteoporosis, or skeletal malformations (scoliosis, hip dysplasia). Some participants received medication that would be expected to affect HR, either by targeting the sinoatrial node (candesartan, ramipril, amlodipine), or by blunting emotional responses (e.g. benzodiazepines) or pain perception (paracetamol, antiepileptic drugs).

### HR monitoring

An increase in HR was detected in specific situations in 13 out of 14 participants (Table 2). This was the case even in those participants whose medication could be expected to affect HR. The three fully ambulant participants were all monitored during seated activities with few postural changes and little muscle work. Increased HR that was interpreted to reflect pain or discomfort in specific situations was detected in 11 out of 14 participants. However, only in six out of the 11 participants (55%) was the HR increase fully reproducible in the sense that HR increased each time a presumed stressful situation arose. In five participants for whom there were available data on HR in specific situations, reproducibility varied from 44 to 93%. In one participant, no HR increase could be identified in any of the situations that were analyzed, and for one participant data on reproducibility were not available.

**Table 2:** Heart rate (HR) observations.

Patient	Typical mean HR ± SD (bpm)	Typical HR increase (bpm)	Hours registered	Increased HR in typical situations	No. of replications of increased HR/No. of measurements
F59	89 ± 7	> 96	51	Physiotherapy Blood glucose testing	72/164 (44%) Not counted
F31	72 ± 4	> 76	29	Fits of tremor and sweating	8/8 (100%)
F18	87 ± 10	> 97	15	During daily nail filing	11/11 (100%)
M54	82 ± 7	> 89	96	Day care center attendance Dressing neuropathic feet	17/17 (100%) Not counted
F58	67 ± 4	> 71	22	Being looked at by children	5/5 (100%)
M62	73 ± 6	> 79	42	No findings	0
F 36	85 ± 11	> 96	115	The sight of apparatus used for standing upright Transport in patient lift	11/11 (100%) Not counted
M48*	78 ± 7	> 85	2:45	Extension of spastic arm	25/27 (93%)
M 37*	58 ± 10	> 68	9	The sight of apparatus used for standing upright Transport in patient lift	10/22 (45%) Not counted
F56	64 ± 12	> 76	11	Physiotherapy (joyful)	14/14 (100%)
M 27	91 ± 15	> 106	41	Travel by car (joyful) Epileptic seizures	Not counted 3/3 (100%)
F22	78 ± 11	> 89	150	Going outdoors (stressful)	16/23 (70%)
F14	92 ± 9	> 101	220	Extension of spastic arm Change of diapers Transport in patient lift	22/29 (76%) 19/24 (79%) Not counted
M18	63 ± 11	> 74	110	Watching childrens’ movies Extension of spastic arm	8/9 (89%) 6/6 (100%)

Typical mean HR in beats per minute (bpm) and 1 standard deviation (SD) for each participant. The mean HR, SD and HR increase (> mean HR+ 1SD) were calculated daily. Typical situations that were associated with increased HR are given together with number of replications/total number of observed situations. Eight participants were interpreted as experiencing pain or distress during episodes with increased HR; three participants (F56, M27, and M18) were considered to experience joy during some of the episodes with increased HR.

### Specific and novel findings during HR monitoring

HR increased in several situations that were not previously thought to entail pain or distress by caregivers. In three participants, extension of a spastic arm during dressing or physiotherapy caused increased HR (Table 2). In one of these cases (M48; Tables 2 and 3), this realization led to treatment with botulinum toxin of the elbow flexors, which allowed the participant to extend his arm voluntarily when being dressed.

**Table 3:** Results of HR monitoring.

Patient	Change of practice/improved understanding of the participant?
F59	Reassurance during transportation in patient lift^a^
F31	None (fits of tremor and sweating confirmed to be fear-related)
F18	Moisturizing of nails prior to filing^a^ Physiotherapy not confirmed to cause discomfort
M54	Attendance at day care center understood to be stressful
F58	Understood to be afraid of children
M62	None
F 36	Physiotherapy not confirmed to cause discomfort
M48	Treatment of spastic arm with botulinum toxin^a^
M 37	Reassurance during transportation in patient lift^a^
F56	Physiotherapy not confirmed to cause discomfort
M 27	More joyful car trips are being arranged^a^
F22	Longer preparation and support ahead of, and during, going outdoors^a^
F14	More extensive physiotherapy^a^
M18	Change of daily stretching exercises^a^

For 12 of the 14 non-communicating patients with ID in this study, HR monitoring led to an improved understanding of their preferences, stressors, and dislikes. For eight participants (^a^) there was a change in caregivers’ practice after HR monitoring.

Four participants had increased HR at the sight of an electric patient lift used for their transportation, e.g. from bed to a chair. (Tables 2 and 3). In the lift, the patient hangs from a sling that may swing rather freely. A fifth participant (F31), who had fits of tremor and sweating in various situations, had two of these during transportation in a patient lift. It was discovered that she had fallen out of the lift and onto the floor some months previously. Two participants, who were not ambulant (F36 and M37), had increased HR at the sight of an apparatus used for helping them stand upright. One of these (F36) was assumed to react with joy at the sight of the equipment, but it was discovered that the apparatus was wrongly adjusted so that it likely had caused discomfort or pain.

One participant had increased HR when being looked at by children, e.g. in shopping malls, a finding that was interpreted to reflect anxiety. One participant had increased HR during generalized epileptic seizures, which was interpreted to reflect the seizure rather than an emotional response.

Three participants had HR increases that were interpreted to reflect joy. In one, the HR increase that occurred during physiotherapy was accompanied by laughter; in another the HR increase occurred during car travels or when his grandmother came to visit. A third participant had increased HR when he watched children’s movies.

### Change of care practice following HR monitoring

For eight participants, HR monitoring led to a change in caregivers’ practice (Table 3), including reassurance strategies during transportation in patient lifts, changes in the execution of physiotherapy and daily stretching exercises, and changes in a daily nail filing procedure for a participant who was prone to self-harm by scratching herself. For three participants, HR monitoring did not support the caregivers’ impression that physiotherapy was painful, allowing for more extensive physiotherapeutic procedures. For all but two participants, the use of HR monitoring led to some change in caregivers’ understanding of the participant.

## Discussion

This real-world proof of concept study shows that HR monitoring may identify situations that entail acute pain or discomfort in the daily lives of non-communicating persons with ID. Examples of such situations were passive extension of a spastic arm or transportation in a patient lift. Previous studies have found that caregivers that only use observation of behavior to identify pain, may fail to identify painful conditions in non-communicating persons with ID^17, 27, 28^. HR monitoring may improve this situation.

Increased HR may be caused by both adverse and pleasant stimuli^29–33^. This was illustrated in the present study by participants whose HR increased in response to situations that were likely to be painful and by participants whose HR increased during activities they probably enjoyed. Therefore, a HR increase has to be interpreted in light of its context. Such interpretation is indispensable and at the same time a potential source of error. Erroneous interpretation was illustrated in the present study by the participant who displayed increased HR when mounted in an apparatus used to helping her stand upright: her HR increase was interpreted by caregivers as reflecting joy until it was found that the apparatus was wrongly adjusted and probably caused discomfort or pain. In the present study, HR monitoring revealed not only pain, but also distress. Some of these distress reactions, e.g. to nail filing or being looked at by children, were rather idiosyncratic in nature. HR monitoring has to be combined with careful, long-term observation to correctly identify such person-specific stressors. Similarly, HR may increase as an emotional reaction to certain caregivers, rather than to the procedures they perform. Again, only careful observation may distinguish between the two possibilities.

At present, there is no gold standard for identifying pain in non-communicating persons with ID. In an experimental study in which non-communicating persons with ID received pressure-induced pain, Benromano et al. found facial expression, monitored by a video camera and analyzed retrospectively, to be a more reliable indicator of pain than HR^19^. In the present study, however, only some of the participants had intelligible facial expression. Further, the use of video monitoring would have been difficult in our study, which took place in the daily lives of the participants in their communal residences or day care centers. At present, therefore, we believe that HR monitoring is a promising mode of identifying at least some acutely painful or distressing situations that non-communicating persons with ID experience.

When, in the present study, we understood increased HR to be an indicator of pain or distress, this was presumed to be acute pain or distress^29–31^. Data are scarce on HR during prolonged or chronic pain, but some clinical studies suggest that prolonged pain is not accompanied by increased HR^34, 35^. These findings point to the need to find parameters other than HR to identify long lasting pain in non-communicating persons with ID. In future studies, several approaches may prove useful in detecting pain and distress in non-communicating persons, including monitoring electrodermal activity, cortisol levels in saliva, movement with actigraphy, and heart rate variability.

### Limitations

In the present study we used a definition of increased HR (HR that persisted > 5 s at 1 SD above the daily mean) that allowed for individual and day-to-day variations. This definition is arbitrary, however, and we may have failed to detect some cases of increased HR due to the definition being too strict. Further, although we found that HR increased in situations that could be expected to be stressful, we do not know our rates of false positive or false negative results. In some participants, HR did not increase consistently in situations that were interpreted as being stressful. This finding may reflect the occurrence of false negative results due to our definition of increased HR.

Autonomic responses to distress are modified by predictability: unpredicted distress causes stronger autonomic responses^36^. We assume that non-communicating persons with ID are prone to experiencing distress as unpredictable because of their limited understanding of the necessity for painful procedures, such as physiotherapy for spasticity or pinpricks for blood glucose monitoring. We further assume that their reduced ability to avoid distress or to notify their caregivers about it makes stressful situations even more distressing than would be expected in the general population. Therefore, it is possible in the present study that the increases in HR that we detected in presumed distressing situations reflected some combination of pain and the fear that the pain provoked. We are at present not able to distinguish between the two.

HR is expected to increase in response to postural changes or muscle work, which could interfere with the interpretation of increased HR as a sign of distress. In the present study, however, such factors were probably not an important source of error, because HR monitoring predominantly took place when participants were seated or lying down, and because the occurrence of postural changes and muscle work was included in the contextual interpretation of HR variations. For instance, the increase in HR that was seen in several participants in transportation situations occurred at the mere sight of a patient lift, indicating an anticipatory reaction rather than a response to postural change.

In the present study, HR increases could be detected in participants irrespective of age, sex, or medication. However, these factors could impact the degree of HR increase, as previously shown in other patient populations^30, 31^, affecting the sensitivity of HR monitoring and reproducibility of results.

We did not monitor HR after the introduction of changes to care practice, although doing so could have shed light on the validity of HR monitoring as a means of identifying acute pain or distress. Moreover, it could have shed light on whether the changes in care practice reduced distress or not. Therefore, studies with longer term HR monitoring are needed.

## Methods

### Recruitment of participants

The study was approved by The Committee for Ethics in Medical Research for the Southern and Eastern parts of Norway (Concession #2016/1956) and conformed to the Declaration of Helsinki^37^. It was first posted at ClinicalTrials.gov Dec. 13, 2019 (identifier: NCT04199299).

**Figure 1:**
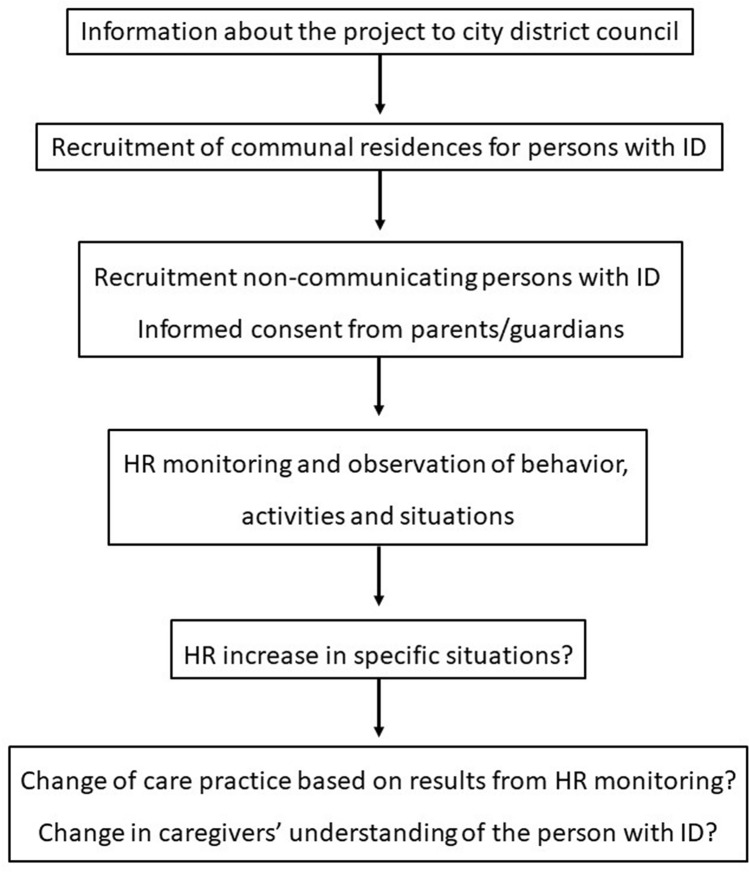
Flow chart of the logic of the study: the recruitment of non-communicating persons with ID, the monitoring of their HR to identify situations that may cause pain or distress, and the resulting change in care practice or in caregivers’ understanding of the non-communicating persons with ID.

A group of 14 adults with intellectual disability who lived permanently in communal residences was recruited in the following manner (Fig. 1): The investigators presented the study to the local administrations of two districts of Oslo city, Norway, which contacted the leaders of the districts’ communal residences for persons with ID. Professional caregivers at the communal residences approached the parents or wardens of potential participants with written material describing the study. Informed, written consent was obtained from parents or legally authorized representative of participants. All parents or legal representatives that were invited agreed to the participation. None of the participants withdrew from the study. Prior to HR monitoring, full-time caregivers working in the communal residences and with good knowledge of the participants were asked to complete the Inventory of potential communicative acts questionnaire (IPCA) about how the participant conveyed being happy, sad, bored, amused, frightened, in pain, angry, or tired^24^.

The cognitive function of all participants was individually assessed by a licensed neuropsychologist (KS). Adults with profound or severe ID are estimated to have a mental age below 6 years of age^8^, and psychological tests that cover this age range were therefore chosen. Participants were assessed with tasks from Bayley scales of infant and toddler development, third edition^38^, which is normed up to 3 years and 5 months of age, and with tasks from the Wechsler preschool and primary scale of intelligence, fourth edition^39^, which is normed for the age range 2 years and 6 months to 7 years and 7 months. For the participants with the most severe motor impairments, tasks could be answered using eye gaze technology. This enabled participants to respond regardless of their speech and motor impairments. Such adaptations have previously been shown not to influence test results^40^.

### HR monitoring

Garmin HRM4 (Olathe, Kansas, USA) chest straps were used to monitor HR. The chest strap detects electric signals from the heart, which are transmitted to a Garmin Forerunner 235 wristwatch. The HR signals were transferred from the wristwatch to a personal computer and converted to spreadsheet format with a program produced in house using GPS Babel as transformation software (see supplementary material).

The participants engaged in varied daily activities, including alternating between staying in their communal residences and visiting day care centers or their family home. Therefore, HR monitoring had to be done as was practically feasible. HR monitoring took place during weekdays. Most participants had the chest strap put in place during dressing in the morning, while some participants had it put on in the afternoon when they returned from day care center. The participants did not sleep with the HR monitor on.

In some participants, the chest strap monitors sometimes lost contact with the skin, so that the electric signals from the heart were lost. This was especially the case in participants with scoliosis or spastic cerebral palsy (Table 1). When loss of contact was discovered the chest strap was immediately put back in place to avoid loss of data.

### Definition of HR increase

An increase in HR was defined as HR that persisted > 5 s at 1 standard deviation (SD) above the mean HR, which was calculated after completion of each HR monitoring session. This definition allowed for individual and day-to-day variations in HR.

### Registration of behaviors, activities, and situations

The study period was 2 months. Participants were accompanied 1:1 by professional caregivers or students of social education. Students of social education (~ 20–50 years old) were completing a 3-year full-time study at Oslo Metropolitan University. Caregivers or students registered behaviors (e.g. certain movements, grimacing, screams, laughter), activities that the participant took part in on a regular basis (e.g. physiotherapy sessions, meals, naps, swimming), and various situations that arose (e.g. visit from a relative, choking on food during a meal). The registered behaviors, activities, and situations were aligned with HR data retrospectively. Therefore, increased HR did not cause caregivers to intervene in real time. Because the study period was limited (2 months), HR was not monitored after care practice was changed.

## Supplementary Information

Supplementary Information.

## Data Availability

Raw data will be supplied upon request.
