# Supplementary material for: Increased heart rate functions as a signal of acute distress in non-communicating persons with intellectual disability
Source: Sci Rep. 2021 Mar 19;11:6479. doi: 10.1038/s41598-021-86023-6 (PMC7979830; doi:10.1038/s41598-021-86023-6)
Supplement: Supplementary file 1 — Supplementary Information. [file 41598_2021_86023_MOESM1_ESM.docx]

**SUPPLEMENTARY MATERIAL**

**Increased heart rate functions as a signal of acute distress in non-communicating persons with intellectual disability.**

Emilie Kildal,^1^ Kristine Stadskleiv,^2,3^ Elin S. Boysen,^4^ Tone Øderud,^4^ Inger-Lise Dahl,^5^ Trine M. Seeberg, ^4^ Svein Guldal,^6^  Frode Strisland,^4^ Cecilie Morland,^7,8^ Bjørnar Hassel^1,9^

1: Department of Neurohabilitation, Oslo University Hospital, Oslo, Norway

2: Department of Clinical Neurosciences for Children, Oslo University Hospital, Oslo, Norway

3: Department of Special Needs Education, University of Oslo, Oslo, Norway

4: SINTEF Digital, Oslo, Norway

5: Oslo Municipality, Nordstrand, Oslo, Norway

6: Norwegian Farmers’ Association, Oslo, Norway

7: Oslo Metropolitan University, Department of Behavioral Science, Oslo, Norway

8: Department of Pharmacy, University of Oslo, Oslo, Norway

9: Institute of Clinical Medicine, University of Oslo, Oslo, Norway

Corresponding author: Bjørnar Hassel, Department of Neurohabilitation, Oslo University Hospital, Oslo, Norway

Tel: +4722118080; e-mail: bjornar.hassel@medisin.uio.no

**Transformation of HR data from a chest belt HR sensor to Excel format**

For each HR observation period, the HR detected via the Garmin HRM4 chest strap was stored as a Garmin .fit file on the GARMIN Forerunner wristwatch. Regularly, all new .fit files were transferred to a personal computer via the Forerunner USB data/charging cable.

Excel as a tool for initial analyses

To be able to analyze the HR data, the data in the .fit files had to be transformed to a readable format. Microsoft Excel was chosen as a manageable tool. To ensure that all caregivers combined their observations of the user’s activities and behaviors with the recorded HR in the same manner, we developed a program that creates an Excel-file containing the recorded HR data, templates for registering observations and graphs that combine the HR and observations. The program lets the caregivers choose their preferred .fit-file (Figure 1) and a name for the resulting file.


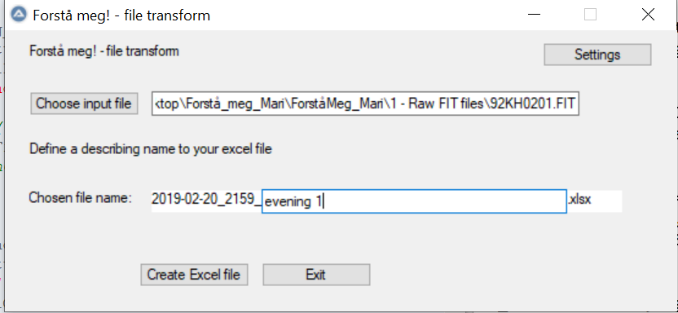


Figure 1. Graphical user interface that allows the user to choose which file to transform to Excel-format (.xlsx) and to give it a name

Then, the script automatically creates an Excel file with four sheets:

- Sheet 1 is for charting activities (e.g. meals, transportation, or physiotherapy sessions) and behaviors (e.g. screams or movements) and when they occurred (Figure 2.)


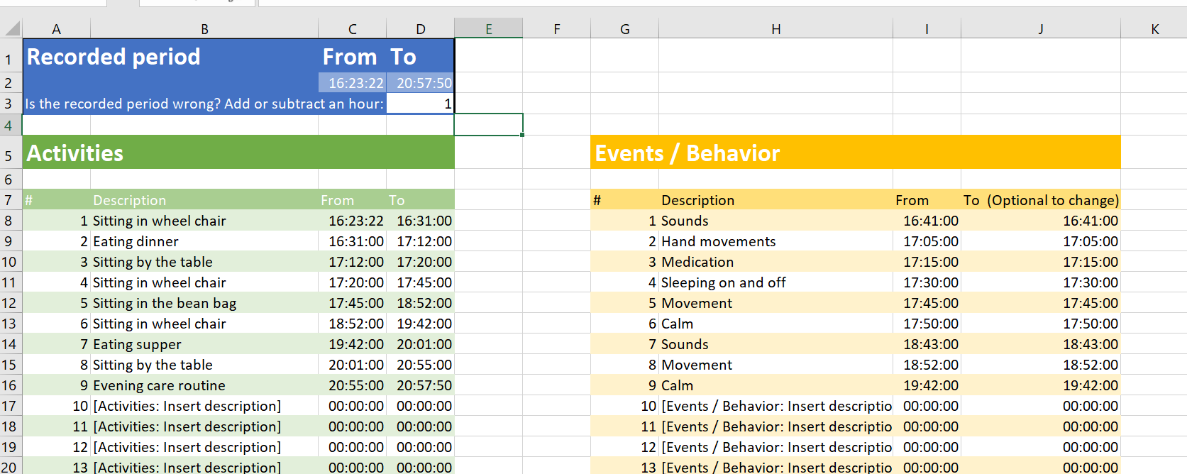


Figure 2: Microsoft Excel sheet for registering observations of activities and behaviors

- Sheet 2 shows a graph of the whole HR recording period with a summary of the average HR in the period, the average plus 1 standard deviation (SD) and the average plus 2 SD (Figure 3).

Behavior

Activities

HR graph (dark blue dots/line)

Average (black line)

Average + 1 SD (pink line)

Average + 2 SD (blue line)


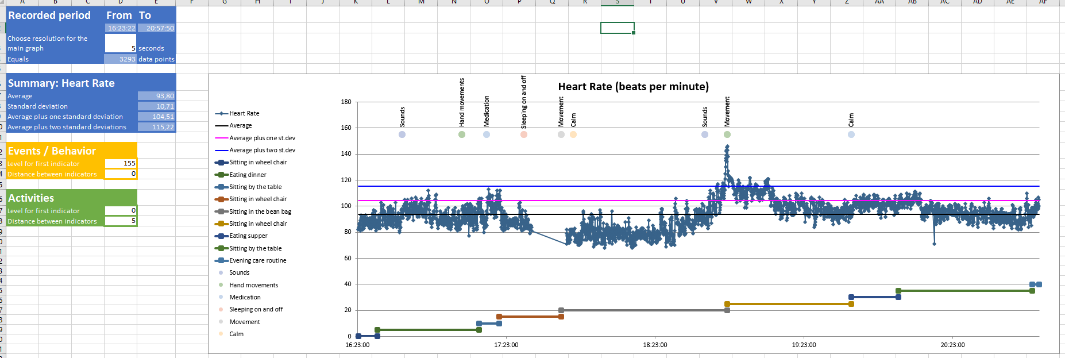


Figure 3: Microsoft Excel sheet showing HR data and observations for the whole registration period

Also, all events and activities described on the observation Excel sheet can be shown in the graph along with the HR.

- Sheet 3 allows the caregiver to select a shorter section of the recorded period, where the average HR and SD are shown for the selected time period. All relevant observations (behaviors and activities) in the selected time range can also be shown (Figure 4).


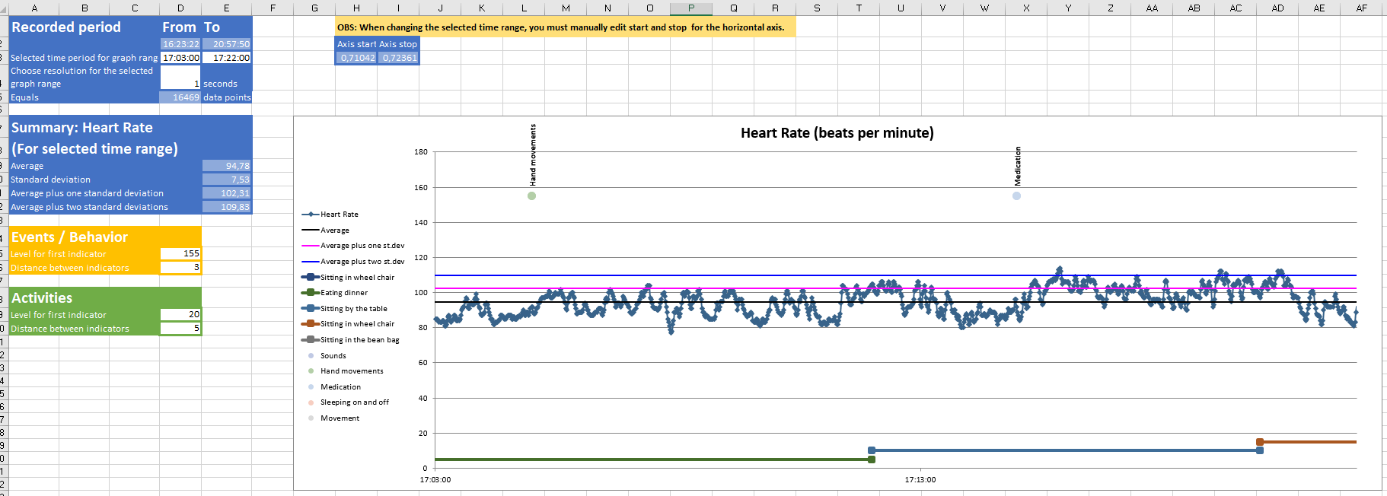


Figure 4: Microsoft Excel sheet showing HR data and observations for a selected time range in the registration period

- Sheet 4 shows the raw data: time and HR values and a set of derived values and formulas forming the bases for the previous sheets (Figure 5).


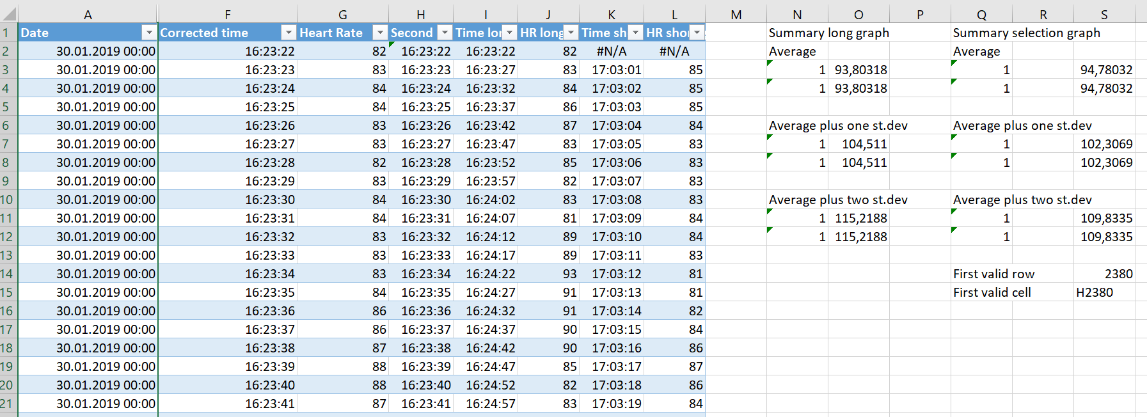


Figure 5: Microsoft Excel sheet for viewing raw data and calculations based on user input in sheets 1-3

Generating the Excel file

The program that creates the Excel template is developed using AutoIt v3. AutoIt v3 is a freeware scripting language designed for automating the Windows graphic user interface and general scripting. AutoIt v3 packages are available for download from <https://www.autoitscript.com/>.

We wrote a script using the tools available from autoitscript.com and compiled to an executable (.exe) referred to as "the program" in the previous and further description. The program was installed on the PCs of each of the caregivers along with the program GPS Babel. GPSBabel <https://www.gpsbabel.org/> is a software free to use it within terms of the GNU Public License that converts data from the .fit format used by Garmin to a more easily readable XML-file.

Our program (both script source code and an executable) is available for download from <https://github.com/SINTEF/UnderstandHowIAm>.

Whether run directly form the script or from a compiled executable file (.exe), our program will do the following steps:

INITIALIZING

- Check if the user has previously given path names for folders where the program will 1) look for .fit files, 2) store intermediate .xml files and 3) store the final Excel-files (.xlsx). If these folders do not exist, the user will be prompted to name paths to them or create them. The paths will be stored in a config file (text file)

- Check whether the path to GPSBabel.exe is given

TRANSFORMING

- Prompt the user to choose the .fit file to be transformed and to give the resulting Excel-file a name

- Run GPSBabel on the selected .fit file and store the resulting .xml file in the designated folder

DATA TRIMMING

- Open the .xml file in Excel and store the file as .xlsx

- Identify columns where HR and time are found

- Remove all data that are not HR or time

CREATING TABLES AND FORMULAE

- Create sheets 1-4 as described above

- Modify the sheet that contains HR and time with formulae necessary to create charts

To calculate the average HR, the standard Excel formula AVERAGE(...) was used over the range of all the HR values. The standard deviation was calculated using the formula STDEV.P(...) over the same range. For the graph where a specific time range was selected, the same formulae were used, but only over HR values in the selected time range.
